# The impact of elevated CO_2_ on methanogen abundance and methane emissions in terrestrial ecosystems: A meta-analysis

**DOI:** 10.1016/j.isci.2024.111504

**Published:** 2024-11-29

**Authors:** Yiwen Ding, Mingyu Wang, Xiaojuan Du, Xue He, Tianle Xu, Xinyu Liu, Fuqiang Song

**Affiliations:** 1 Engineering Research Center of Agricultural Microbiology Technology, Ministry of Education & Heilongjiang Provincial Key Laboratory of Ecological Restoration and Resource Utilization for Cold Region & Key Laboratory of Microbiology, College of Heilongjiang Province & School of Life Sciences, Heilongjiang University, Harbin 150080, China; 2 Heilongjiang Academic of Forestry Qiqihar Branch, Qiqihar 161005, China

**Keywords:** Earth sciences, Atmospheric science, Earth-surface processes, Environmental science, Biogeoscience

## Abstract

Methane (CH_4_), one of the major greenhouse gases, plays a pivotal role in global climate change. Elevated CO_2_ concentration (eCO_2_) increases soil carbon storage, which may provide a valuable material base for soil methanogenic microorganisms and stimulating their growth, thereby ultimately affecting CH_4_ emissions. Therefore, to comprehend the effect of eCO_2_ on CH_4_ emissions, we conducted a meta-analysis encompassing 398 datasets from 59 publications (total of 50 sample sites). The results show that eCO_2_ promotes both the abundance of the functional methanogenic gene *mcrA* and CH_4_ emissions. However, this enhancement is modulated by a range of factors, such as the eCO_2_ duration, land use types and soil texture, and there are significant interactions. This study offers new insights into the effects of eCO_2_ on CH_4_ emissions across diverse ecosystems and the underlying driving forces, vital for predicting the response of global terrestrial ecosystems in the face of future climate change.

## Highlights

• eCO_2_ significantly increased the abundance of *mcrA* gene and CH_4_ emission
• The promotion of eCO_2_ was only significant in paddy fields
• Effect values derived using a hierarchical mixed-effects meta-analysis model
• The promotion of eCO_2_ was influenced by a variety of moderators

## Graphical abstract

**Figure:**
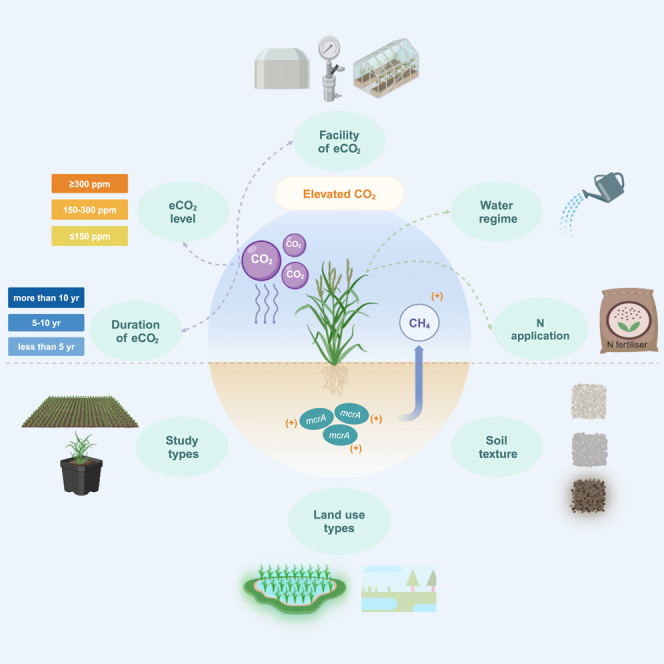

## Introduction

Carbon dioxide (CO_2_) and methane (CH_4_) are the two primary greenhouse gases, accounting for more than 90% of the total radiative forcing exerted by all greenhouse gases.^1^ Anthropogenic activities have driven a surge in atmospheric CO_2_ concentrations, which have escalated from 280 parts per million (ppm) at the onset of the Industrial Revolution to approximately 420 ppm today, marking an unprecedented level over the past two million years.^2^^,^^3^^,^^4^^,^^5^ Concurrently, the global atmospheric concentration of CH_4_ has been soaring, currently reaching 1,853 parts per billion (ppb), representing a remarkable 157% increase compared to pre-industrial levels.^6^^,^^7^^,^^8^ Although atmospheric CH_4_ concentrations are considerably lower than those of CO_2_, it has been estimated that, in the short term, the greenhouse effect caused by CH_4_ is approximately 28 times higher than that of CO_2_.^9^^,^^10^ Even more concerning is the potential for elevated atmospheric CO_2_ concentrations (eCO_2_) to stimulate significant CH_4_ emissions from terrestrial ecosystems.^11^^,^^12^ Previous studies have found that eCO_2_ promotes rice growth, increasing the number of tillers and aerenchyma formation, thereby facilitating enhanced CH_4_ transport and emission.^13^

As a crucial raw material for plant photosynthesis, eCO_2_ plays a pivotal role in promoting plant growth and biomass accumulation. This not only increases the carbon (C) content within plants but also alters soil microbial community structures and interspecies interactions.^14^^,^^15^^,^^16^ Accompanied by the input of extra carbon sources during this process, certain specific microbial groups, such as methanogens, may gradually become dominant, leading to substantial production and CH_4_ emission.^17^^,^^18^ Numerous studies have shown that CH_4_ flux, particularly in natural environments, is primarily regulated by two key microbial functional groups: methanogens (microorganisms that produce methane) and methanotrophs (microorganisms that consume methane).^19^^,^^20^ Typically, methanogens produce CH_4_ during the ongoing process of organic matter decomposition, while methanotrophs simultaneously utilize CH_4_, facilitating the CH_4_ cycle mediated by these two microbial functional groups.^21^ In studies exploring CH_4_ metabolism, the functional genes *mcrA* and *pmoA* are widely utilized as markers for methanogens and methanotrophs, respectively. Their combined response to eCO_2_ determines the dynamic characteristics of CH_4_ emissions.^22^^,^^23^^,^^24^ Wang et al. (2023) found that the abundance of the *mcrA* gene in soil increased with eCO_2_ concentrations during different developmental stages of rice, further predicting the potential for CH_4_ emissions.^25^ However, the current limited research is insufficient to precisely elucidate how eCO_2_ stimulate CH_4_ emissions and the abundance of *mcrA* and *pmoA* genes. Therefore, a meta-analysis is necessary to quantify the impact of eCO_2_ on CH_4_ emissions and the abundance of the *mcrA* and *pmoA* genes, and to ascertain the key factors that regulate these impacts.

**Figure 1:**
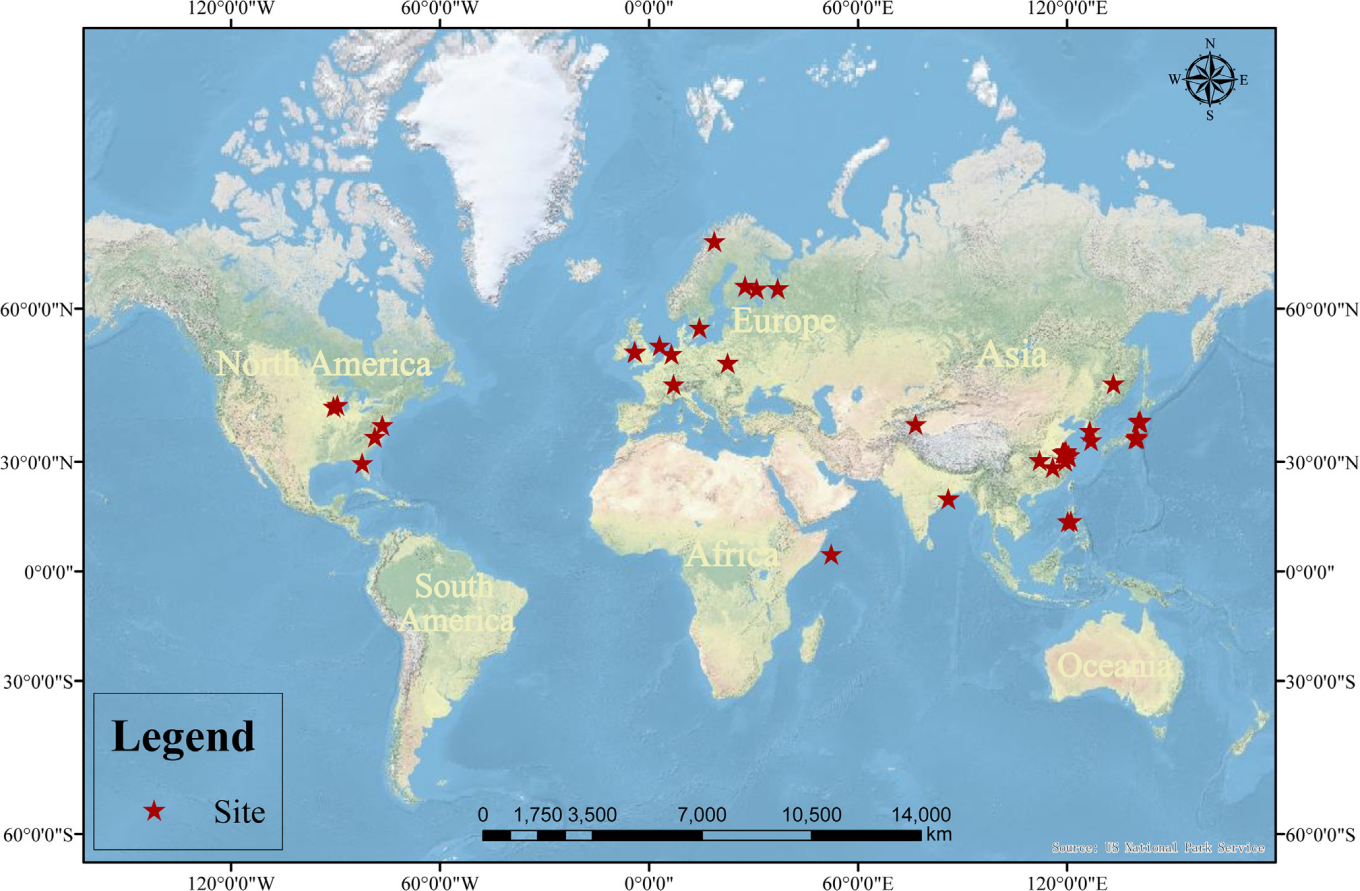
Global distribution of study sites in the publications included in the meta-analyses

Here, we collected data from previous studies on the effects of eCO_2_ on the abundance of the *mcrA* and *pmoA* genes and CH_4_ emissions for meta-analysis, combining 398 datasets from 59 publications (total of 50 sample sites) (Figure 1). The aim is to systematically and quantitatively analyze the effects of eCO_2_ on the abundance of the *mcrA* and *pmoA* functional genes as well as on CH_4_ emissions under different conditions. Meanwhile, our study seeks to establish and corroborate the relationship between these two CH_4_ metabolism functional genes with CH_4_ emissions. Moreover, we identified the effects of eight moderators on CH_4_ emissions under eCO_2_ conditions. We hypothesized that eCO_2_ would increase the abundance of the *mcrA* gene, a methanogenesis-related functional gene, and promote CH_4_ emissions, especially in flooded anaerobic rice ecosystems; and that factors across land use types would have different effects on the responses of the abundance the *mcrA* and *pmoA* genes and CH_4_ emissions under eCO_2_ conditions. This study aims to accurately assess the magnitude of the impact of eCO_2_ on CH_4_ emissions and the functional genes associated with methane metabolism, as well as to evaluate the relative importance of the moderators modulating these impacts. This will contribute to predicting the response of terrestrial ecosystems to future climate change, which is crucial for sustainable land management, natural resource conservation and maintenance of ecological balance.

## Result

### Effect of eCO_2_ on the abundance of functional methane metabolism genes *mcrA* and *pmoA*

**Figure 2:**
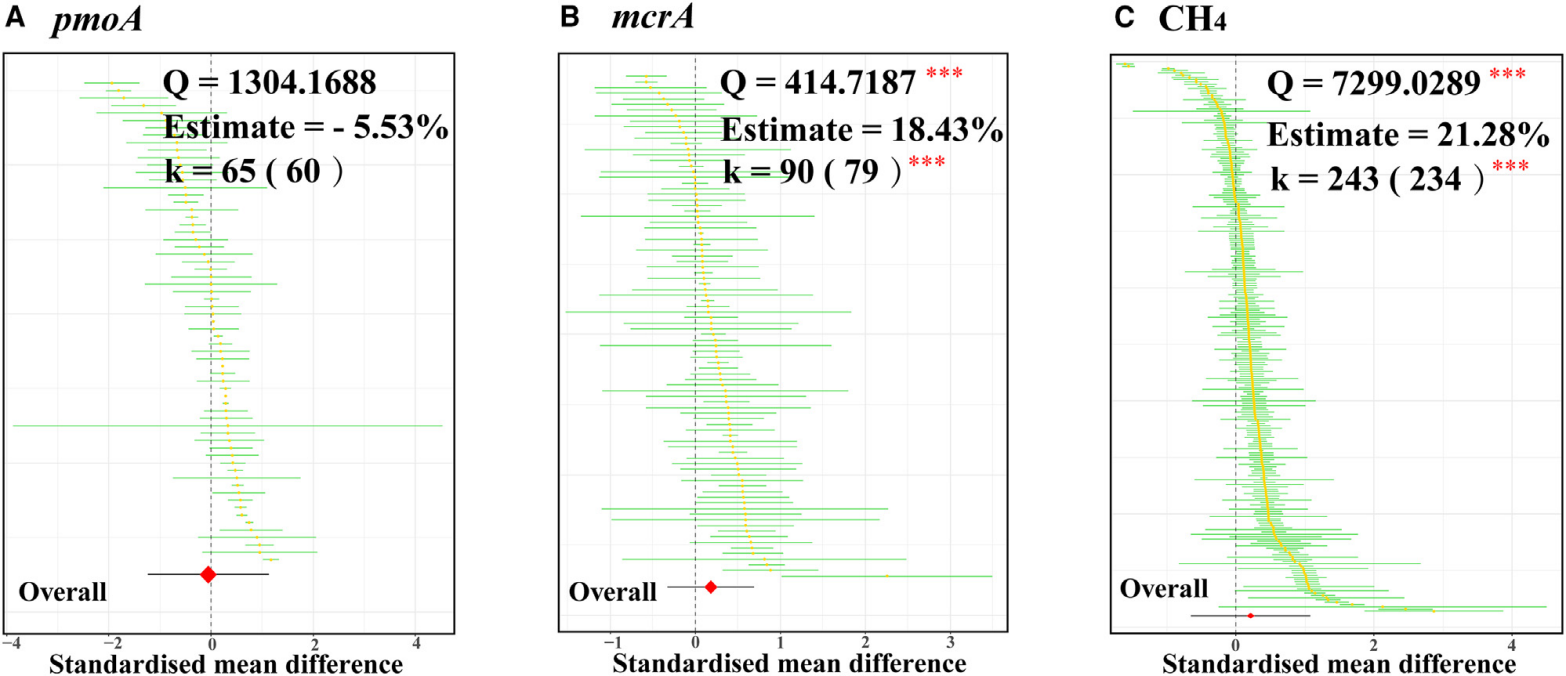
The cumulative effect sizes of eCO_2_ on methane oxidation functional gene *pmoA*, methanogenesis functional gene *mcrA* and CH_4_ emissions Catterpillar plots of the effect size estimates for eCO_2_ on functional gene *pmoA* (A), *mcrA* (B), and CH_4_ emissions (C), the number of observations is beside each attribute without parentheses, and the number of studies is in parentheses (k). Log response ratios (effect sizes) are shown as yellow dots with 95% confidence intervals (CI 95%) as green lines. The overall weighted mean effect size estimate is shown as a red diamond at the bottom, with the prediction interval as a black line.

Overall, eCO_2_ had no significant effect on the abundance of the *pmoA* gene (Figure 2A, *p* > 0.05) but significantly increased the abundance of the *mcrA* gene (Figure 2B, *p* < 0.001). The overall weighted mean effect size estimate was higher than zero but with greater overall heterogeneity (Q = 414.7, *p* < 0.001) (Figure 2B). To explain this heterogeneity, we introduced the following eight moderators to explain this heterogeneity: land use types, duration of eCO_2_, eCO_2_ level, facility of eCO_2_, nitrogen (N) fertilizer application concentration, water regime, soil texture, and study type. The points in the model formed a funnel plot symmetric around the mean effect by the Egger regression test (Figure S1, *p* > 0.05), indicating that our model had no publication bias or heterogeneity.

**Figure 3:**
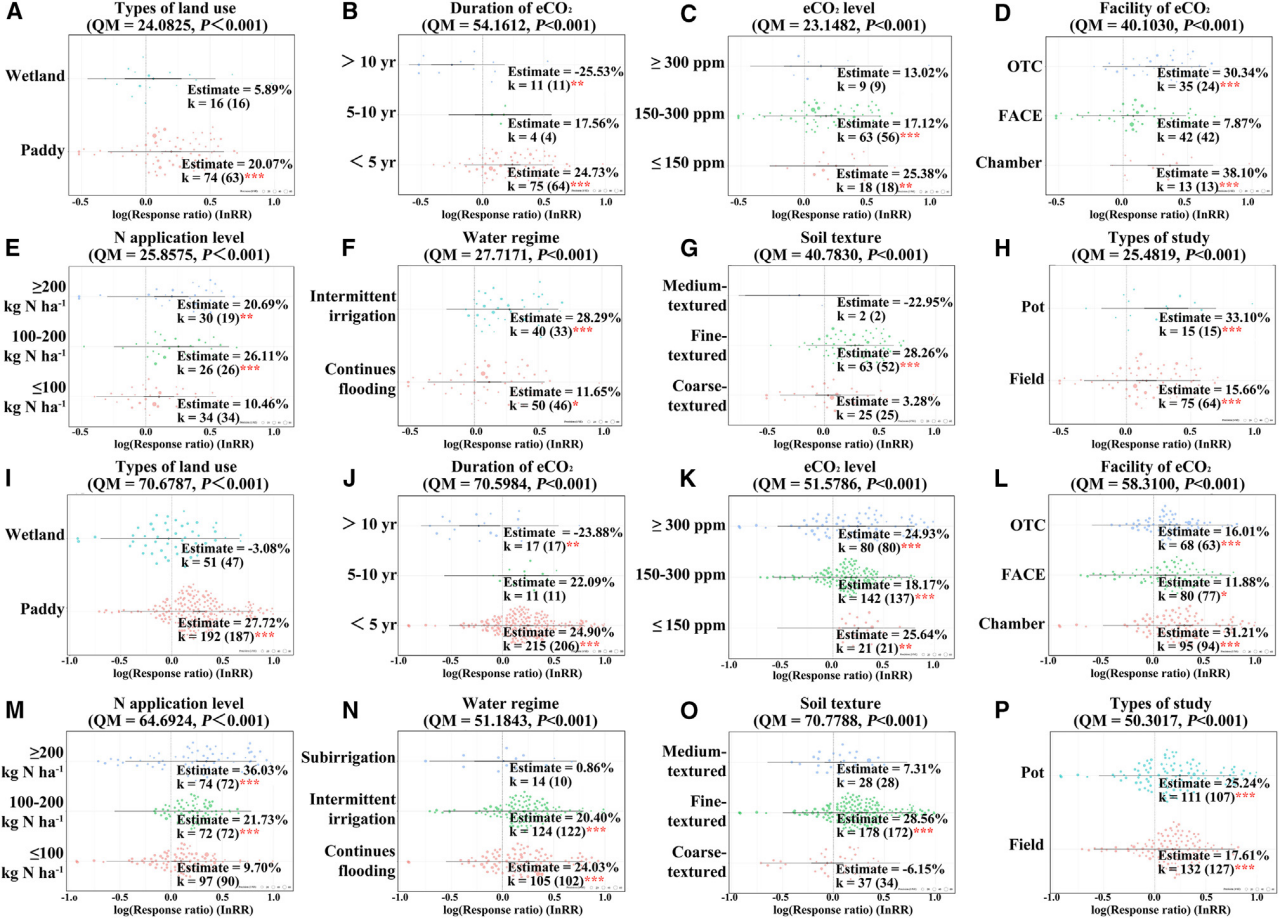
Meta-analysis of the effect of eCO_2_ on methanogenesis functional gene *mcrA* and CH_4_ emissions across different factors (A–P) Orchard plots show the mean estimates of the effect of eCO_2_ on the methanogenesis functional gene *mcrA* (A–H) and CH_4_ emissions (I–P) under the influence of different factors (open circles), with individual effect sizes (colored circles) for the different factors. The precision (inverse variance) is plotted as a thick line at CI 95%, with the prediction interval as a thin line. The significance tests are presented by *p* values, the number of observations is beside each attribute without parentheses, and the number of studies is in parentheses (k). (A and I): types of land use, (B and J): duration of eCO_2_, (C and K): eCO_2_ level, (D and L): facility of eCO_2_, (E and M): N application level, (F and N): water regime, (G and O): soil texture, and (H and P): types of study.

Under different land use types, there were some differences in the response of the abundance of the *mcrA* gene to eCO_2_ (Qm = 24.1, *p* < 0.001) (Figure 3A). In the paddy fields, eCO_2_ led to a significant increase in the abundance of the *mcrA* gene (*p* < 0.001, 27.72%); however, no such significant change was observed in the wetland (*p* > 0.05) (Figure 3A).

Among the moderators associated with eCO_2_, when eCO_2_ duration less than 5 years the strongest positive effect (24.73%) on the abundance of the *mcrA* gene was observed; whereas eCO_2_ duration more than 10 years had a significant negative effect (−25.53%) (Figure 3B). For eCO_2_ experimental facility, the elevating effect of eCO_2_ on the abundance of the *mcrA* gene was stronger in the growth chambers (38.10%) as compared to Open Top Chamber (OTC) (30.34%) (Figure 3D).

The effect (lnRR) of eCO_2_ on the abundance of the *mcrA* gene was also affected by different N fertilizer application concentrations (Qm = 25.9, *p* < 0.001) and water regime conditions (Qm = 27.7, *p* < 0.001) (Figures 3E and 3F). Notably, when N fertilizer application was less than 100 kg ha^−1^ eCO_2_ had no significant effect on the abundance of the *mcrA* gene (*p* > 0.05) (Figure 3E).

Furthermore, the extent of this effect varies significantly across different soil textures (Qm = 40.8, *p* < 0.001) and study types (Qm = 25.5, *p* < 0.001). The effect of eCO_2_ on the abundance of the *mcrA* gene was only significant in fine-textured soils (28.26%) (Figure 3G); additionally, a notable stimulatory effect was also observed under pot experiment conditions (33.10%) (Figure 3H).

### Effect of eCO_2_ on CH_4_ emissions

eCO_2_ significantly increased CH_4_ emissions (*p* < 0.001); however, the overall heterogeneity was large (Q = 7299.0, *p* < 0.001, Figure 2C), which was similar to the response of the abundance of the *mcrA* gene to eCO_2_ (Figure 2B). The contribution of eCO_2_ to CH_4_ emissions (27.72%) was significant only in paddy fields (Qm = 70.7, *p* < 0.001), and not in wetlands (*p* > 0.05) (Figure 3I).

In terms of eCO_2_ duration (Qm = 70.6, *p* < 0.001), there was a positive effect of eCO_2_ on CH_4_ emissions (24.90%) when the duration was less than 5 years; however, interestingly, eCO_2_ duration more than 10 years suppressed CH_4_ emissions (−23.88%) (Figure 3J). In addition, the response of CH_4_ emissions to eCO_2_ was significantly influenced at different eCO_2_ levels (Qm = 51.6, *p* < 0.001) and eCO_2_ facilities (Qm = 58.3, *p* < 0.001). In the condition of eCO_2_ ≤150 ppm (25.64%) and under growth chamber (31.21%), eCO_2_ had the strongest contribution to CH_4_ emission (Figures 3K and 3L).

It is noteworthy that eCO_2_ has varying degrees of effect on CH_4_ emissions under different water and fertilizer management practices. For example, eCO_2_ had the strongest promotion when N fertilizer was applied at a concentration of ≥200 kg ha^−1^ (36.03%) (Figure 3M); and it was higher under continuous flooding (24.03%) than intermittent irrigation (20.40%) (Figure 3N).

The promotion of CH_4_ emissions by eCO_2_ was more significant only in fine textured soil (28.56%) (Figure 3O); and in the pot experiment (25.24%) eCO_2_ had the strongest effect (Figure 3P).

### Relative contributions of the moderators on the observed effects

**Figure 4:**
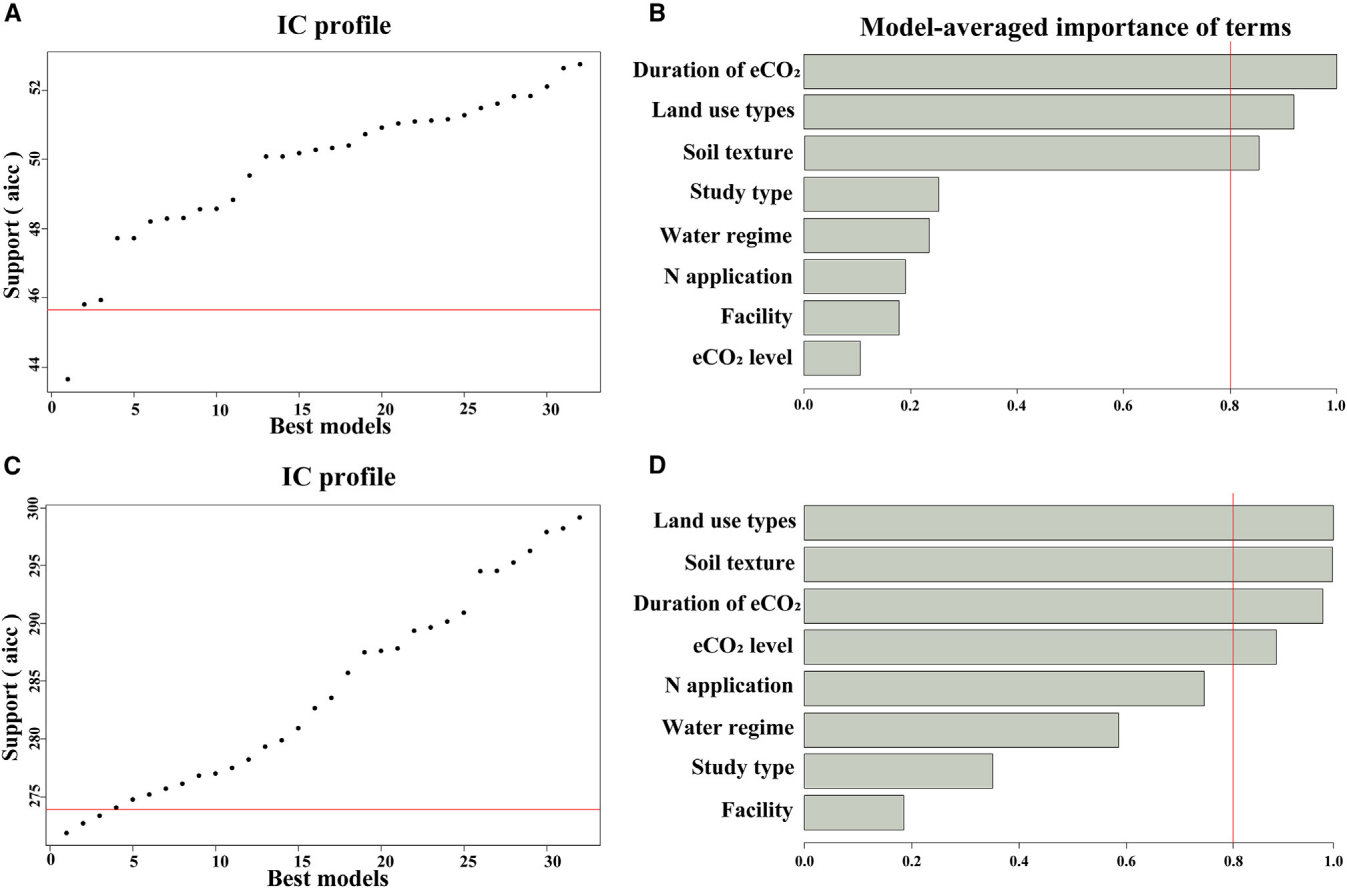
Importance of duration of eCO_2_, land use types, soil texture, type of study, water regime, N application, eCO_2_ facility, and eCO_2_ level on *mcrA* gene copy numbers and CH_4_ emissions under eCO_2_ (A–D) (A) (for *mcrA* gene copy numbers) and figure (C) (for CH_4_ emissions) respects the importance of the moderators with IC profile (A and C). The importance on *mcrA* gene copy numbers (red line in B) and CH_4_ emissions (red line in D) is based on the sum of Akaike weights derived from model selection (B and D).

We used a random forest model to predict the relative importance of different moderators that increased the abundance of the *mcrA* gene and CH_4_ emissions under eCO_2_, and the smaller Akaike Information Criterion (AIC) values indicated a better fit of the random forest model (Figures 4A and 4C). Under eCO_2_, the effects of eCO_2_ duration, land use types, and soil texture played a key role in the abundance of the *mcrA* gene (Figure 4B). The duration of eCO_2_, land use types, and soil texture are among the more critical moderators that modulate the effect of eCO_2_ on the abundance of the *mcrA* gene. The effects of eCO_2_ on CH_4_ emissions were mainly moderated by land use types, soil texture, eCO_2_ duration, and eCO_2_ level (Figure 4D).

**Figure 5:**
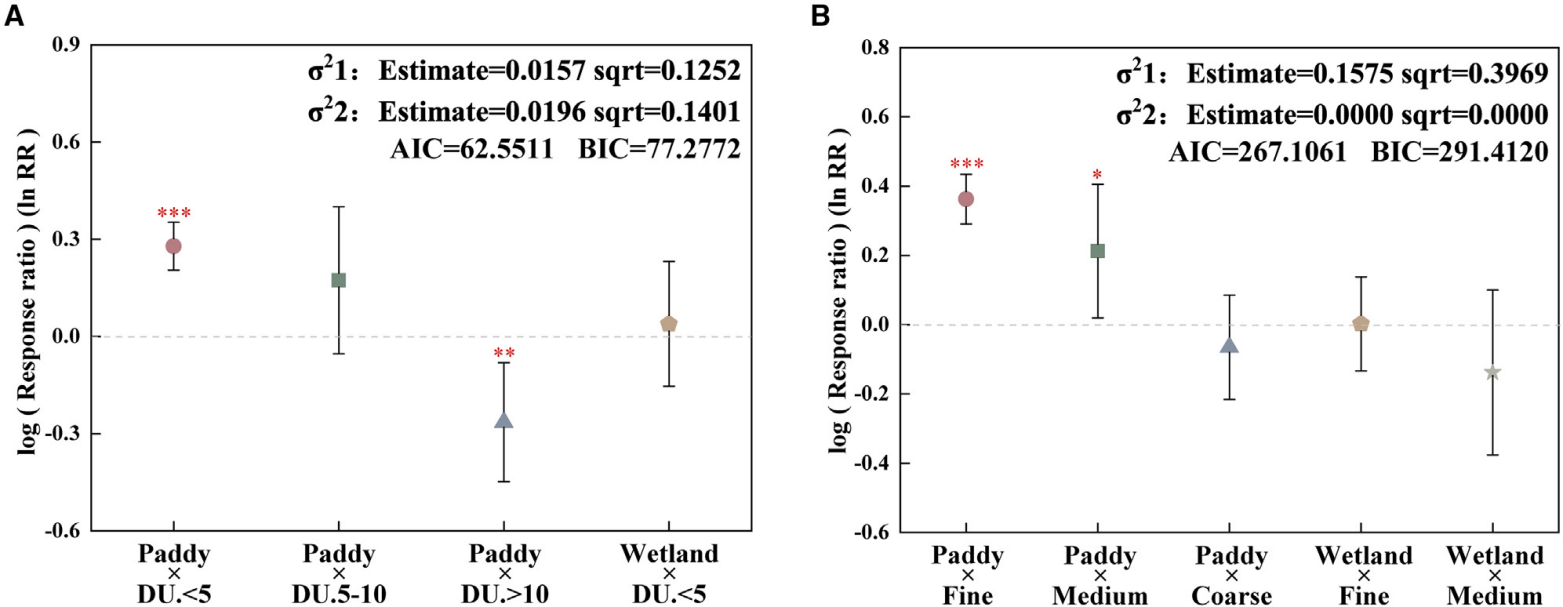
Meta-analysis of the effect on *mcrA* gene copy numbers and CH_4_ emissions under eCO_2_ across two-factor interaction The data represent weighted means and their corresponding 95% confidence intervals for the lnRR. An impact of two-factor interaction is considered present when the confidence interval does not intersect the zero line. Results are not presented when the sample size is lower than 3. (A) lnRR of *mcrA* gene copy numbers across interaction of land use type and eCO_2_ duration. (B) lnRR of CH_4_ emissions across interaction of landuse type and soil texture. DU.＜5 = eCO_2_ duration less than 5 years; DU.5–10 = eCO_2_ duration 5–10 years; DU.＞10 = eCO_2_ duration more than 10 years; fine, fine-textured soil; medium, medium-textured soil; coarse, coarse-textured soil.

To analyze the interaction between the moderators, we conducted a two-way ANOVA on the top two most important factors predicted by the random forest model (Figure 5). In the paddy field, the promotion effect of eCO_2_ on the abundance of the *mcrA* gene was superimposed when eCO_2_ duration was less than 5 years (*p* < 0.001); however, the effect of eCO_2_ on the abundance of the *mcrA* gene became inhibitory under the interaction of eCO_2_ duration more than 10 years and paddy (*p* < 0.01) (Figure 5A). For CH_4_ emissions, the significant stimulating effects of superposition between soil texture and land use type were observed only in paddy fields with fine-textured soils (*p* < 0.001) and paddy fields with medium-textured soils (*p* < 0.05) (Figure 5B).

## Discussion

### Effect of eCO_2_ on the abundance of functional methane metabolism genes *mcrA* and *pmoA*

eCO_2_ has been widely documented to exert profound impacts on the characteristics and functions of terrestrial ecosystems.^26^ The escalating concentrations of CO_2_ not only provide an enhanced substrate for CH_4_ emissions within terrestrial ecosystems but also augment plant-derived soil C inputs, subsequently exerting indirect effects on soil microbial community structure and function.^27^^,^^28^ Methanogens and methanotrophs, the two primary functional microbial groups, involved in the CH_4_ cycle, play crucial roles in this process. Methanogens, ancient bacteria widely distributed in anaerobic environments, convert organic matter into CH_4_ in the final step of anaerobic degradation.^29^ Methanotrophs, on the other hand, utilize CH_4_ as their primary energy source for both assimilative and dissimilative metabolism.^30^ Consequently, the responses of these two functional microbial groups to eCO_2_ may ultimately result in alterations in CH_4_ emissions.

Methanogens possess a unique enzyme, methyl-coenzyme M reductase, encoded by the *mcrA* gene, which catalyzes the final step in CH_4_ production.^31^ The abundance of functional genes, serving as a crucial indicator for assessing population size and metabolic activity, is a key focus of our attention regarding its response to eCO_2_.^32^ Our meta-analysis reveals that eCO_2_ stimulates an increase in the abundance of the *mcrA* gene (Figure 2A). Approximately two-thirds of CH_4_ in soil originates from the decomposition of organic matter by methanogens under anaerobic conditions, with acetate serving as the primary C source ultimately converted into CH_4_.^33^^,^^34^ While the rise in atmospheric CO_2_, a major contributor to global climate change, may pose significant environmental challenges, numerous studies have confirmed that eCO_2_ can introduce substantial amounts of C into terrestrial ecosystems.^35^ Given that soil microbiota represents a major group metabolizing C sources, their active response to eCO_2_ is inevitable.^36^ This explains why eCO_2_ significantly stimulates an increase in the abundance of the *mcrA* gene.

The particulate CH_4_ monooxygenase, encoded by the *pmoA* gene in methanotrophs, oxidizes CH_4_ into methanol, playing a crucial role in reducing CH_4_ emissions from terrestrial ecosystems.^37^^,^^38^ Notably, methanotrophs typically consume 60%–80% of the CH_4_ produced by methanogens, helping to mitigate excessive CH_4_ emissions from the soil.^39^^,^^40^ Generally speaking, an increase in the CH_4_ emission is bound to promote the development of methanotrophs; however, in our research findings, the abundance of the *pmoA* gene did not increase in parallel with the abundance of the *mcrA* gene.^35^ This unexpected result may be attributed to the characteristics of the CH_4_ monooxygenase encoded by the *pmoA* gene. Due to its low affinity, this enzyme requires high concentrations of CH_4_ in the soil for robust development.^41^ However, the emission of CH_4_ caused by eCO_2_ is a slow process. For methanotrophs, this lag in CH_4_ exposure may obscure a more pronounced response of the abundance of the *pmoA* gene to eCO_2_.^42^ As CH_4_ serves as the sole C source for methanotrophs, their growth demands a high concentration of this substrate. The increased CH_4_ emissions resulting from eCO_2_ may not be sufficient to stimulate significant growth of methane-oxidizing flora.^43^

The comprehensive meta-analysis results indicate significant heterogeneity in the promotion of *mcrA* gene expression by eCO_2_. To decompose this overall heterogeneity, we have incorporated several important explanatory variables, including land use types, eCO_2_ duration, eCO_2_ level, eCO_2_ facility, and management practices. Previous research has demonstrated that eCO_2_ in agroecosystems elevates the copy number of the *mcrA* gene in rice root systems.^44^ This is supported by the result of our meta-analysis that eCO_2_ increased the abundance of *mcrA* significantly only in paddy fields (Figure 3A). As predicted by the random forest model, land use type is a relatively important factor influencing the abundance of *mcrA* gene under eCO_2_ (Figure 4B). eCO_2_ usually leads to an adequate supply of C in the soil, enabling methanogens to obtain more organic C.^45^ Consequently, this enhances their physiological activities by boosting respiration and regulating nutrient cycling. At the same time, rice paddy soils have high soil moisture content, resulting in inundated conditions that are conducive to anaerobic methanogens.^46^ These conditions may stimulate methanogens activity, thereby increasing the abundance of related functional genes.

Delving deeper into the factors regulating this effect, we found that low concentrations of eCO_2_ (eCO_2_ ≤ 150 ppm) had the greatest promotion on the abundance of the *mcrA* gene (Figure 3C), whereas low concentrations of N fertilizer application did not significantly affect the abundance of the *mcrA* gene (Figure 3E). This result may be attributed to the ability of low concentrations of eCO_2_ to stimulate root growth and increase root exudation, thereby enhancing the potential C source for root-associated microorganisms and consequently increasing the abundance and activity of root-associated methanogens.^13^^,^^47^ In contrast, high concentrations of eCO_2_ (eCO_2_ ≥ 300 ppm) induce hypoxic soil conditions, where soil oxygenation regulates CH_4_ oxidation to a greater extent.^48^^,^^49^ This shift could potentially inhibit methanogen activity. Moreover, during rice growth stages, under eCO_2_ the depletion of available N leads to higher C/N ratios, limiting the decomposition of organic matter (e.g., detached roots and root exudates). This results in a reduction of methanogenic substrates and their utilization by functional microorganisms.^50^^,^^51^ Therefore, low concentrations of N fertilizer application may offset this part of impact on methanogens and not significantly affect the abundance of the *mcrA* gene under eCO_2_.

In addition, according to the random forest model, we predicted eCO_2_ duration and land use type are the two most important explanatory variables for the abundance of the *mcrA* gene under eCO_2_ (Figure 4B). Further, a two-way ANOVA using a mixed-effects model on these two variables found that short-term eCO_2_ (eCO_2_ duration less than 5 years) increased the abundance of the *mcrA* gene in paddy fields; however, this effect reversed to a negative effect after eCO_2_ duration more than 10 years (Figure 5A). It has been shown that methanotrophs possess superior ecotolerance, which allows them to survive over a wider range of pH and temperature conditions.^52^ In contrast, methanogens exhibit less environmentally adaptable, and require suitable environment for growth and metabolism.^13^ However, long-term eCO_2_ will alter soil properties, which may inhibit the physiological activity of methanogens to some extent and reduce the abundance of the *mcrA* gene.

Overall, eCO_2_ significantly promoted the abundance of the *mcrA* gene and this process was regulated by a variety of moderators. Based on this, subsequent studies ought to focus on the effects of eCO_2_ on the abundance of the *mcrA* gene, taking into account the effects of external conditions and the intricate interplay among them.

### Effect of eCO_2_ on CH_4_ emissions

Amid global climate changes, particularly the enrichment of atmospheric CO_2_, the concentrations of atmospheric CH_4_ have been consistently rising at an annual rate ranging from 1.0% to 1.2%.^53^^,^^54^^,^^55^ In a changing world, sustained eCO_2_ is a very thorny issue, with concentrations rising until 2100.^56^ Therefore, a more accurate understanding of the impact of eCO_2_ on CH_4_ emissions will be better achieved by exploring the moderators of this process.

The results of our meta-analysis indicated that eCO_2_ significantly promoted CH_4_ emissions from paddy fields (Figure 3C), which could also be corroborated with the increase in the abundance of the *mcrA* gene (Figure 3A). Similar to the result of random forest predictions, land use type is also a relatively important factor for CH_4_ emissions under eCO_2_ (Figure 4D). It has been shown that eCO_2_ may indirectly increase CH_4_ emissions by promoting net primary production.^57^ Furthermore, we found that the stimulatory effects of eCO_2_ on both the abundance of the *mcrA* gene and CH_4_ emissions were most pronounced in fine-textured soils (Figures 3G and 3O). More importantly, through the random forest model we have predicted that soil texture serves as a significantly moderator in regulating both the abundance of the *mcrA* gene and CH_4_ emissions under eCO_2_ (Figures 4B and 4D). In fact, fine-textured soils are more suitable for rice cultivation with better aeration, permeability, water retention and heat insulation.^58^^,^^59^ The results of our two-way analysis of variance (ANOVA) further support this finding, indicating that the promotive effect of eCO_2_ on CH_4_ emissions is most evident under the combined influence of paddy fields and fine-textured soil (Figure 5B). The mechanism behind this is that eCO_2_ stimulates converting enzymes to degrade more sucrose in the rice inter-root,^60^^,^^61^^,^^62^ which provides sufficient substrate C for methanogens and promotes the activity of methanogens, thereby increasing CH_4_ emissions.^63^^,^^64^

The application of N fertilizers serves as a pivotal factor influencing CH_4_ emissions, thereby inevitably shaping the response of CH_4_ emissions to eCO_2_. The reported effects of N fertilizers on CH_4_ emission are complex and sometimes contradictory. They depend on the nature of the fertilizer, the quantity applied, and the method of application.^65^ We analyzed and concluded that the application of N fertilizer amplifies the promotion of eCO_2_ to CH_4_ emissions (Figure 3M). Our analysis was confirmed by Zhang et al. (2016)^66^ N application stimulated CH_4_ production more than CH_4_ oxidation resulting in net CH_4_ emission from N-fertilization treatments. Because soil nutrient content changes due to large inputs of exogenous nutrient elements, which in turn affects CH_4_ metabolism cycles and inhibits the farmland’s CH_4_ oxidation process and promote CH_4_ emission.^67^^,^^68^^,^^69^

Moreover, our results showed the contribution of eCO_2_ to CH_4_ emissions is stronger in closed environments such as pot experiments and growth chambers (Figures 3L and 3P). This may be due to the higher sensitivity of closed ecosystems to fluctuations in CO_2_ concentration.^49^ Specifically, the spatial confines of the plant-soil system are relatively small under pot experiment conditions, where changes in the concentration of eCO_2_ may be more readily perceived and responded to by microorganisms and plants.^70^ Then increased the abundance of the *mcrA* gene and close activity of methanogens favoring the promotion of methane production.^71^ On the other hand, the environment of growth chambers is usually stable and closed, which allows experimental conditions to be more precisely controlled from other external conditions.^72^^,^^73^ Meanwhile, this study showed that the enhanced effect of eCO_2_ on CH_4_ emissions was slightly lower under intermittent irrigation than under continuous flooding (Figure 3N). The physicochemical processes and successive steps involved in the establishment of anaerobiosis, which enable methanogenesis after submersion, are widely known.^74^ When the soil is submerged, dissolved O_2_ concentration rapidly decreases and facultative anaerobic, then microaerophilic, and finally strict anaerobic microorganisms develop. Under the condition of continuous flooding soil, anaerobic methanogens in the soil remain persistently active, which contributes to the generation and emission of CH_4_.^40^

In conclusion, our study highlights the complex relationship between eCO_2_ and factors influencing CH_4_ emissions from paddy fields. Future studies should synthesize the interactions between different environmental factors and their combined effects on CH_4_ production and emissions in the context of climate change.

### Conclusion

Amid the global backdrop of continuously rising CO_2_ levels, the responses of the functional gene *mcrA* involved in CH_4_ metabolism in soil and CH_4_ emissions to eCO_2_ offer new insights into our understanding of the methane cycle. The response of the abundance of the *mcrA* gene and CH_4_ emission to eCO_2_ were consistently promoted by eCO_2_, and the promotion of both by eCO_2_ was influenced by a variety of moderators. Among them, the duration of eCO_2_, land use types, and soil texture were more crucial in the promotion of the abundance of *mcrA* gene and CH_4_ emission by eCO_2_. And there is some interaction between the effects produced by the factors. Despite the limited data available for inclusion in the current meta-analysis, our findings emphasize that under eCO_2_ the ability of terrestrial ecosystems to act as sinks for mitigating warming may be considerably offset by the increase in the intensity of the soil Greenhouse Gas (GHG) sources they induce.

### Limitations of the study

Our meta-analysis found a consistent pattern of the abundance of the *mcrA* gene and CH_4_ emissions in response to eCO_2_, i.e., eCO_2_ increased both. Although this meta-analysis was fruitful in exploring the effects of eCO_2_ on CH_4_ emissions from wetlands and rice paddies, there are still obvious limitations. Firstly, we could not further analyze the real reason for the non-significant effect of eCO_2_ on the *pmoA* gene, nor could we confirm whether eCO_2_ has an effect on the CH_4_ oxidation process in which the *pmoA* gene is involved. Because there are few previous studies on the amount of CH_4_ oxidation under eCO_2_ conditions, it was not possible to incorporate and analyze the available data. Second, although the anthropogenic eCO_2_ can precisely control the variables, it is difficult to fully simulate the complexity and dynamics of long-term elevation of atmospheric CO_2_ concentration in the natural environment. Complex environmental variables (climate type, geographic location, etc.) and changes in soil physicochemical properties under the long-term effects of eCO_2_ may increase the uncertainty in evaluating the effects of eCO_2_ on CH_4_ emissions.^75^^,^^76^ More importantly, only the link between the copy number of key functional genes and CH_4_ emission was considered, which is relatively singular, ignored the changes in microbial community structure and functional diversity and other microbial indicators.^77^^,^^78^ Thirdly, the distinct ways in which plants conduct photosynthesis dictate their methods of CO_2_ fixation and utilization efficiency. This variation may exert a significant regulatory influence on how eCO_2_ affects CH_4_ emissions. Due to data constraints, we are unable to access a comprehensive dataset for most plant species, which constitutes a limitation in our study. Lastly, elevated CO_2_ concentration would promote forest C cycling by enhancing forest productivity and increasing living biomass stock,^79^ whereas in our study we only focused on the effects of CO_2_ enrichment experiments on CH_4_ emissions in wetlands and rice paddy. Therefore, in order to fully analyze and comprehensively explore the effects of eCO_2_ on CH_4_ emissions in the whole terrestrial ecosystem, it should also not neglect the response of ecosystems such as forests and grasslands to eCO_2_. In future studies, we should extend the timescale of the study and continuously monitor and record the changes of the soil under eCO_2_ conditions. At the same time, combining the experimental data under different ecosystems and adding the indicators such as bacterial community structure and functional diversity, so as to adopt a more comprehensive and systematic approach to elucidate in depth the dynamic effects of eCO_2_ on the CH_4_ emission process.

## Resource availability

### Lead contact

Further information and requests for resources should be directed to and will be fulfilled by the lead contact, Fuqiang Song (0431sfq@163.com).

### Materials availability

The study did not generate any new materials.

### Data and code availability

• All data reported in this paper will be shared by the lead contact upon request.
• This study did not report original code.
• Any additional information required to reanalyze the data reported in this paper is available from the lead contact upon request.

## Acknowledgments

This work was funded by the Heilongjiang Provincial Key R&D Program Projects (GA23B006), Heilongjiang Province “Double-First Class” Discipline Collaborative Innovation Achievement Construction Project (LJGXCG2023-088), and 10.13039/501100001809National Natural Science Foundation of China (31971527).

## Author contributions

Y.D. and M.W. designed the study and prepared the manuscript. X.D. and X.H. conducted the result analysis. T.X. revised this manuscript and language editing. X.L. and F.S. supervised the work and gave guidance. All coauthors contributed to manuscript editing. All authors have read and agreed to the published version of the manuscript.

## Declaration of interests

The authors declare no competing interests.

## STAR★Methods

### Key resources table

**Table:** 

REAGENT or RESOURCE	SOURCE	IDENTIFIER
**Deposited data**		

Web of Science	https://www.webofscience.com/	N/A
Google Scholar	http://scholar.google.com/	N/A

**Software and algorithms**		

EndNote	https://support.clarivate.com/Endnote/s/article/DownloadEndNote?language=en_US	Version 20.0.0.14672
R software	https://www.r-project.org/	Version 4.1.1
Web Plot Digitiser software	https://automeris.io/WebPlotDigitizer/	Version 4.6

### Experimental model and study participant details

Our study does not use experimental models typical in the life sciences.

### Method details

#### Focus of the study

We focused the meta-analysis on terrestrial ecosystems.

#### Systematic literature search

To ensure a systematic and comprehensive literature search, we followed the Preferred Reporting Items for Systematic Reviews and Meta-Analyses (PRISMA) workflow.^80^^,^^81^ A comprehensive literature search were conducted utilizing the search engine Google Scholar and the databases Web of Science. The keywords are listed below: “elevated CO_2_” or “eCO_2_” or “CO_2_ enrichment” or “elevated CO_2_ concentration” or “CH_4_ emissions” or “CH_4_ emissions rate” or “*mcrA*” or “*pmoA*” or “methanogenesis functional gene” or “methane oxidation functional gene” or “methanogens” or “methanotrophs”.

#### Criteria for inclusion of studies

The following criteria for inclusion were used to screen the published literature: (1) only English-language literature; (2) Control and eCO_2_-treatment groups, including at least three replications; (3) data including one of the indicators related to the abundance of *mcrA*, abundance of *pmoA*, and CH_4_ emissions from paddies or wetlands; (4) sample size, mean, standard error (SE), or standard deviation (SD) recorded in tables, graphs, texts, or supplementary materials. Based on the above criteria, we selected 59 publications from around the world and collected 398 datasets from them (Table S1). This meta-analysis included a total of 50 sample sites, mainly in Asia, Europe and North America, as shown in Figure 1.

#### Meta-data extraction

For all articles included in the meta-analysis, we extracted data including: (1) basic information of the included studies, such as title, first author, and publication date; (2) information on the study site, including latitude and longitude; (3) Explanatory variables data (see data grouping), including regulators associated with eCO_2_(eCO_2_ duration, eCO_2_ level, facility of eCO_2_), soil conditions (type of landuse, soil texture), management practices(N application level, water regime), type of study; (4) Experimental data, including *mcrA* gene abundance or *pmoA* gene abundance or CH_4_ emission for both control and eCO_2_-treatment groups, with the corresponding sample size, mean, SE or SD. The data presented in the form of tables were collected directly from the articles. However, if the data were presented in graphical form, they were collected using Web Plot Digitiser software 4.6^82^ to extract data from the graphs. Furthermore, we collected all associated statistical measures to conduct a meta-analysis.

#### Data grouping

To allow cross-comparisons between studies, explanatory variables were grouped based on information published in peer-reviewed articles. The eCO_2_ duration were grouped as less than 5 years, 5–10 years, and more than10 years. The eCO_2_ level were grouped as ≤150 ppm, 150–300 ppm, and ≥300 ppm. The facility of eCO_2_ were grouped as OTC, FACE, and chamber. The types of landuse were grouped as paddy and wetland. The soil texture were grouped as fine-textured, medium-textured, and coarse-textured soils, as defined by Cornell Soil Health Laboratory recommendations.^83^ The fine-textured group was composed of clay loams, silty clay loams, sandy clays, silty clays, and clays. The medium textured group was composed of loams, silt loams, silts, and sandy clay loams, while the coarse-textured group included sands, loamy sands, and sandy loams. The N application level were grouped as ≤100 kg ha^−1^, 100–200 kg ha^−1^, and ≥200 kg ha^−1^. The water regime were grouped as continuous flooding, intermittent irrigation, and subirrigation. The types of study were grouped as field and pot.

#### Meta-analysis

In this meta-analysis, the natural log response ratio (ln RR) was used as an effect value to calculate the effect of eCO_2_ on the abundance of methanogenesis functional gene *mcrA*, methane oxidation functional gene *pmoA,* and CH_4_ emissions.^84^ The effect sizes for lnRR were calculated as follows:

lnRR=ln(Xt/Xc)=lnXt−lnXc

where ln RR values indicate whether variables (e.g., abundance of *mcrA*, abundance of *pmoA*, and CH_4_ emissions) are positively or negatively affected by eCO_2_, respectively, and where *Xt* and *Xc* are the mean values of the target variables in the control and treatment groups, respectively. The variance (v) of each single effect size was calculated according to Hedges et al. (2010) as follows:^85^

$$v=\frac{St^{2}}{n_{t}Xt^{2}}+\frac{Sc^{2}}{n_{c}Xc^{2}}$$

where *n*_*t*_ indicates the number of treatment samples and *n*_*c*_ indicates the number of control samples. *St* is the standard deviation (SD) of the treatment and *Sc* is the standard deviation of the control group. When only the standard error (SE) was provided in the reported data, the standard deviation (SD) of the control and treatment groups was calculated by SD = SE × √n. When standard deviation (SD) and standard error (SE) were missing, the "imput_SD" function in the "metagear" package on the R 4.1.3 software was used to fill in the missing standard deviation (SD) values in the collated data.^86^^,^^87^ The weights of the effect values were calculated by taking the inverse of the sampling variance (W_ij_):

$$W_{\text{ij}}=\frac{1}{vi+tau^{2}}$$

where W_*ij*_ is the weight associated with each lnRR observation, *tau*^2^ is the between-case variance, and *vi* is the within-case variance. We used the following weighted response rates (Ln RR_++_) to determine the overall effect of the eCO_2_ treatment and the control, calculated as follows:

$$\mathrm{Ln}\;{\text{RR}}_{++}=\frac{\sum \frac{m}{i=1}\sum \frac{k}{j=1}W_{ij}LnRR_{ij}}{\sum \frac{m}{i=1}\sum \frac{k}{j=1}W_{ij}}$$

The SE of Ln RR_++_ is calculated using the following equation:

$$S\left(\mathrm{Ln}\;{\text{RR}}_{++}\right)=\sqrt{\frac{1}{\sum \frac{m}{i=1}\sum \frac{k}{j=1}W_{ij}}}$$

where m is the number of groups, k is the number in group i, and W_ij_ is the weight of each lnRR_++_. Positive and negative values of ln RR_++_ indicate positive and negative effects, respectively.

95% confidence intervals (95% CI) were calculated using the following formula:

$$95\%\text{Cl}=\mathrm{Ln}\;{\text{RR}}_{++}\pm 1.96•S\left(\mathrm{Ln}\;{\text{RR}}_{++}\right)$$

If the 95% confidence interval does not intersect 0, eCO_2_ is significantly correlated with the abundance of the methanogenesis functional gene *mcrA*, methane oxidation functional gene *pmoA,* and CH_4_ emissions; the opposite (95% confidence interval intersects 0) represents no statistical significance.

When the heterogeneity is too large, we use mixed-effects models to analyze which potential factors cause this heterogeneity. The analyzed dataset was based on a stratified structure (Multiple treatment groups correspond to one control group) in order to obtain more accurate and reliable results, a hierarchical mixed effects meta-analysis model was applied to account for differences in different study cases.^88^ However, extracting multiple effect sizes from the same publication may violate the assumption of effect size independence. To minimize potential data non-independence, we systematically assigned number to each dataset, ensuring that distinct numbers correspond to data from different publications, while data from the same publication share the same number.^89^^,^^90^^,^^91^^,^^92^ For each observation, we calculated the effect size (ln RR) using the "escalc" function in the "metafor" package on the R software.^93^ The effect size observation (ln RR) was calculated using the "escalc" function in the "metafor" package.^94^ Hierarchical random effects models were set up by means of the "rma.mv" function in the "metafor" package.^94^ We assessed the heterogeneity of effect sizes through Cochran’s Q-test (*Qt*).^95^ The larger the value of *Qt*, the higher the data heterogeneity and the greater the dispersion. We divide overall heterogeneity (*Qt*) into heterogeneity due to known factors (*Qm*) and heterogeneity due to unknown factors (*Qe*):

Qt=Qm+Qe

where the *Qm* value can reflect the magnitude of the influence of the explanatory variables on the effect value. We used *Qm* and significance tests (*p*-values) as quantitative metrics to determine the extent to which eCO_2_ influenced the abundance of methanogenesis functional gene *mcrA*, methane oxidation functional gene *pmoA,* and CH_4_ emissions. In addition, *Qm* values and significance tests (*p*-values) can be used as quantitative metrics to determine the response extent on the abundance of methanogenesis functional gene *mcrA*, methane oxidation functional gene *pmoA,* and CH_4_ emissions to eCO_2_.

#### Assessment of risk of bias

In traditional meta-analysis, the reliability of the results can be quantitatively tested by means of funnel plots, Egger tests, and insecurity coefficients,^96^^,^^97^ but stratified mixed-effects meta-analysis models constructed under the "rma.mv" function in the "metafor" package results can only be used for model diagnostics in the form of funnel plots and cannot be quantitatively tested by the regtest or ranktest functions. Therefore, we reconstructed a random-effects model using the "rma.mv" function in the "metafor" package for the quantitative test of preference. When the *p*-value of the model results is greater than 0.05, it indicates that the funnel shape is symmetrical, implying that the study results are relatively unaffected by publication bias (Figure S1).^98^^,^^99^

### Quantification and statistical analysis

All raw data were collated and initially processed by Excel 2019 (Microsoft, Seattle, WA, USA). Data were analyzed using the "metafor" package in the R4.2.1 software.^93^ We used the "Orchard" package in the R software to visualize the effect of eCO_2_ on methanogenesis functional gene *mcrA*, methane oxidation functional gene *pmoA,* and CH_4_ emissions, with Orchard displaying effect sizes, 95% confidence intervals, and prediction intervals.^100^ Random forest models were constructed using the "randomForest" package in the R software^101^ to analyze the significance of the different moderators on the effect of eCO_2_ on the abundance of methanogenesis functional gene *mcrA*, methane oxidation functional gene *pmoA* and CH_4_ emissions (with a threshold of 0.8). Statistical model selection was performed using the "glmulti" package in the R software. The base model was first constructed using the "rma.glmulti" function, then the hierarchical model and all the moderators were defined, and the different models were systematically evaluated and compared with the Aicc values to obtain the model that best fits the data.^102^ In the meta-analysis, we conducted a two-way ANOVA (Analysis of Variance) to assess the impact of the interaction between the top two most important moderators predicted by the random forest model on the effect size. In meta-analyses, model diagnostics are crucial.^103^

## Supplemental information

Document S1. Figures S1

Table S1. 398 datasets selected and collected from 59 publications, based on the criteria for inclusion of studies, related to the STAR Methods

## References

[1] Zheng Y., Wang H., Liu Y., Liu P., Zhu B., Zheng Y., Li J., Chistoserdova L., Ren Z.J., Zhao F. (2024). Electrochemically coupled CH_4_ and CO_2_ consumption driven by microbial processes. Nat. Commun..

[2] Xu Z., Jiang Y., Jia B., Zhou G. (2016). Elevated-CO_2_ Response of Stomata and Its Dependence on Environmental Factors. Front. Plant Sci..

[3] The World Meteorological Organization (WMO) (2022). More bad news for the planet: greenhouse gas levels hit new highs. WMO Greenhouse Gas Bull..

[4] NOAA. Carbon cycle greenhouse gases (2022). Earth system research laboratories and global monitoring laboratory. https://gml.noaa.gov/ccgg/trends/.

[5] Peng S., Ciais P., Maignan F., Li W., Chang J., Wang T., Yue C. (2017). Sensitivity of land use change emission estimates to historical land use and land cover mapping. Global Biogeochem. Cycles.

[6] Poulter B., Bousquet P., Canadell J.G., Ciais P., Peregon A., Saunois M., Arora V.K., Beerling D.J., Brovkin V., Jones C.D. (2017). Global wetland contribution to 2000-2012 atmospheric methane growth rate dynamics. Environ. Res. Lett..

[7] Saengkerdsub S., Ricke S.C. (2014). Ecology and characteristics of methanogenic archaea in animals and humans. Crit. Rev. Microbiol..

[8] Saunois M., Bousquet P., Poulter B., Peregon A., Ciais P., Canadell J.G., Dlugokencky E.J., Etiope G., Bastviken D., Houweling S. (2016). The global methane budget 2000-2012. Earth Syst. Sci. Data.

[9] van Groenigen K.J., Osenberg C.W., Hungate B.A. (2011). Increased soil emissions of potent greenhouse gases under increased atmospheric CO_2_. Nature.

[10] Forster P., Hegerl G., Knutti R., Ramaswamy V., Solomon S., Stocker T.F., Stott P., Zwiers F. (2007). Assessing uncertainty in climate simulations. Nat. Clim. Change.

[11] Hungate B.A., Holland E.A., Jackson R.B., Chapin F.S., Mooney H.A., Field C.B. (1997). The fate of carbon in grasslands under carbon dioxide enrichment. Nature.

[12] Xu C., Zhang N., Zhang K., Li S., Xia Q., Xiao J., Liang M., Lei W., He J., Chen G. (2023). Coupled anaerobic methane oxidation and metal reduction in soil under elevated CO_2_. Glob. Chang. Biol..

[13] Yu H., Zhang G., Xia L., Huang Q., Ma J., Zhu C., Shan J., Jiang Q., Zhu J., Smith P. (2022). Elevated CO_2_ does not necessarily enhance greenhouse gas emissions from rice paddies. Sci. Total Environ..

[14] Wang M., Li D., Frey B., Gao D., Liu X., Chen C., Sui X., Li M. (2024). Land use modified impacts of global change factors on soil microbial structure and function: A global hierarchical meta-analysis. Sci. Total Environ..

[15] Long S.P., Ainsworth E.A., Rogers A., Ort D.R. (2004). Rising atmospheric carbon dioxide: plants FACE the future. Annu. Rev. Plant Biol..

[16] Yue K., Fornara D.A., Yang W., Peng Y., Peng C., Liu Z., Wu F. (2017). Influence of multiple global change drivers on terrestrial carbon storage: additive effects are common. Ecol. Lett..

[17] Conrad R. (2007). Advances in Agronomy.

[18] Mosier A.R., Morgan J.A., King J.Y., LeCain D., Milchunas D.G. (2002). Soil-atmosphere exchange of CH_4_, CO_2_, NO_x_, and N_2_O in the Colorado shortgrass steppe under elevated CO_2_. Plant Soil.

[19] Cheng X., Wang H., Zeng Z., Li L., Zhao R., Bodelier P.L.E., Wang Y., Liu X., Su C., Liu S. (2022). Niche differentiation of atmospheric methane-oxidizing bacteria and their community assembly in subsurface karst caves. Environ. Microbiol. Rep..

[20] Cheng X., Zeng Z., Liu X., Li L., Wang H., Zhao R., Bodelier P.L.E., Wang W., Wang Y., Tuovinen O.H. (2023). Methanotrophs dominate methanogens and act as a methane sink in a subterranean karst cave. Sci. Total Environ..

[21] Wang L., Ge J., Feng L., Liu Y., Li Y., Wang J., Xiao X., Zhang Z. (2022). The Synergism between Methanogens and Methanotrophs and the Nature of their Contributions to the Seasonal Variation of Methane Fluxes in a Wetland: The Case of Dajiuhu Subalpine Peatland. Adv. Atmos. Sci..

[22] Danilova O.V., Belova S.E., Gagarinova I.V., Dedysh S.N. (2016). Microbial community composition and methanotroph diversity of a subarctic wetland in Russia. Microbiology.

[23] Dettling M.D., Yavitt J.B., Cadillo-Quiroz H., Sun C., Zinder S.H. (2007). Soil–Methanogen Interactions in Two Peatlands (Bog, Fen) in Central New York State. Geomicrobiol. J..

[24] Yun J., Zhang H., Deng Y., Wang Y. (2015). Aerobic methanotroph diversity in Sanjiang wetland, Northeast China. Microb. Ecol..

[25] Wang Y., Hu Z., He S., Jing Q., Shen L., Liu C., Wu Z., Huang W., Lu G., Cao R. (2023). Linear relationship between CH4 fluxes and atmospheric CO2 concentration levels controlled by rice biomass and soil methanogenic communities. Nutrient Cycl. Agroecosyst..

[26] Liu X., Shen L.D., Yang W.T., Tian M.H., Jin J.H., Yang Y.L., Liu J.Q., Hu Z.H., Wu H.S. (2022). Effect of elevated atmospheric CO_2_ concentration on the activity, abundance and community composition of aerobic methanotrophs in paddy soils. Appl. Soil Ecol..

[27] Bardgett R.D., Wardle D. (2010). Aboveground-belowground linkages: biotic interactions, ecosystem processes and global change. EOS Trans. Am. Geophys..

[28] Butterly C.R., Phillips L.A., Wiltshire J.L., Franks A.E., Armstrong R.D., Chen D., Mele P.M., Tang C. (2016). Long-term effects of elevated CO_2_ on carbon and nitrogen functional capacity of microbial communities in three contrasting soils. Soil Biol. Biochem..

[29] Nayak D.D., Mahanta N., Mitchell D.A., Metcalf W.W. (2017). Post-translational thioamidation of methyl-coenzyme M reductase, a key enzyme in methanogenic and methanotrophic Archaea. Elife.

[30] Ding C., Liu Y., Dumont M.G., Pan H., Zhao K., Li Y., Zhang Q., Luo Y., Jiao S., Di H. (2024). Mean annual precipitation modulates the assembly of high-affinity methanotroph communities and methane oxidation activity across grasslands. Agric. Ecosyst. Environ..

[31] Nwokolo N.L., Enebe M.C. (2022). An insight on the contributions of microbial communities and process parameters in enhancing biogas production. Biomass Convers. Biorefin..

[32] Judd C.R., Koyama A., Simmons M.P., Brewer P., von Fischer J.C. (2016). Co-variation in methanotroph community composition and activity in three temperate grassland soils. Soil Biol. Biochem..

[33] Wang W., Zeng C., Sardans J., Wang C., Tong C., Peñuelas J. (2018). Soil Methane Production, Anaerobic and Aerobic Oxidation in Porewater of Wetland Soils of the Minjiang River Estuarine, China. Wetlands.

[34] Wang Y., Hu Z., Shen L., Liu C., Islam A.R.M.T., Wu Z., Dang H., Chen S. (2021). The process of methanogenesis in paddy fields under different elevated CO_2_ concentrations. Sci. Total Environ..

[35] Nwokolo N.L., Enebe M.C. (2024). Methane production and oxidation—A review on the *pmoA* and *mcrA* genes abundance for understanding the functional potentials of the agricultural soil. Pedosphere.

[36] Stralis-Pavese N., Abell G.C.J., Sessitsch A., Bodrossy L. (2011). Analysis of methanotroph community composition using a *pmoA*-based microbial diagnostic microarray. Nat. Protoc..

[37] Evans P.N., Boyd J.A., Leu A.O., Woodcroft B.J., Parks D.H., Hugenholtz P., Tyson G.W. (2019). An evolving view of methane metabolism in the Archaea. Nat. Rev. Microbiol..

[38] Guerrero-Cruz S., Vaksmaa A., Horn M.A., Niemann H., Pijuan M., Ho A. (2021). Methanotrophs: Discoveries, Environmental Relevance, and a Perspective on Current and Future Applications. Front. Microbiol..

[39] Chen X., Diao H., Wang S., Li H., Wang Z., Shen Y., Degen A.A., Dong K., Wang C. (2023). Plant community mediated methane uptake in response to increasing nitrogen addition level in a saline-alkaline grassland by rhizospheric effects. Geoderma.

[40] Le Mer J., Roger P. (2001). Production, oxidation, emission and consumption of methane by soils: A review. Eur. J. Soil Biol..

[41] Li Q., Peng C., Zhang J., Li Y., Song X. (2021). Nitrogen addition decreases methane uptake caused by methanotroph and methanogen imbalances in a Moso bamboo forest. Sci. Rep..

[42] Lin J.L., Joye S.B., Scholten J.C.M., Schäfer H., McDonald I.R., Murrell J.C. (2005). Analysis of Methane Monooxygenase Genes in Mono Lake Suggests That Increased Methane Oxidation Activity May Correlate with a Change in Methanotroph Community Structure. Appl. Environ. Microbiol..

[43] Kumaresan D., Abell G.C.J., Bodrossy L., Stralis-Pavese N., Murrell J.C. (2009). Spatial and temporal diversity of methanotrophs in a landfill cover soil are differentially related to soil abiotic factors. Environ. Microbiol. Rep..

[44] Okubo T., Liu D., Tsurumaru H., Ikeda S., Asakawa S., Tokida T., Tago K., Hayatsu M., Aoki N., Ishimaru K. (2015). Elevated atmospheric CO_2_ levels affect community structure of rice root-associated bacteria. Front. Microbiol..

[45] Dacey J.W.H., Drake B.G., Klug M.J. (1994). Stimulation of methane emission by carbon dioxide enrichment of marsh vegetation. Nature.

[46] Mclain J.E.T., Ahmann D.M. (2008). Increased moisture and methanogenesis contribute to reduced methane oxidation in elevated CO_2_ soils. Biol. Fertil. Soils.

[47] Das S., Adhya T.K. (2012). Dynamics of methanogenesis and methanotrophy in tropical paddy soils as influenced by elevated CO_2_ and temperature interaction. Soil Biol. Biochem..

[48] Bhattacharyya P., Roy K.S., Neogi S., Manna M.C., Adhya T.K., Rao K.S., Nayak A.K. (2013). Influence of elevated carbon dioxide and temperature on belowground carbon allocation and enzyme activities in tropical flooded soil planted with rice. Environ. Monit. Assess..

[49] Qian H., Chen J., Zhu X., Wang L., Liu Y., Zhang J., Deng A., Song Z., Ding Y., Jiang Y. (2022). Intermittent flooding lowers the impact of elevated atmospheric CO_2_ on CH_4_ emissions from rice paddies. Agric. Ecosyst. Environ..

[50] Bodelier P.L.E., Laanbroek H.J. (2004). Nitrogen as a regulatory factor of methane oxidation in soils and sediments. FEMS Microbiol. Ecol..

[51] Zheng X., Zhou Z., Wang Y., Zhu J., Wang Y., Yue J., Shi Y., Kobayashi K., Inubushi K., Huang Y. (2006). Nitrogen-regulated effects of free-air CO_2_ enrichment on methane emissions from paddy rice fields. Glob. Chang. Biol..

[52] Levine U.Y. (2009).

[53] IPCC (Intergovernmental Panel on Climate Change) (1995). https://www.ipcc.ch/report/ipcc-guidelines-for-national-greenhouse-gas-inventories/.

[54] Sauterey B., Charnay B., Affholder A., Mazevet S., Ferrière R. (2020). Co-evolution of primitive methane-cycling ecosystems and early Earth’s atmosphere and climate. Nat. Commun..

[55] Yvon-Durocher G., Allen A.P., Bastviken D., Conrad R., Gudasz C., St-Pierre A., Thanh-Duc N., del Giorgio P.A. (2014). Methane fluxes show consistent temperature dependence across microbial to ecosystem scales. Nature.

[56] IPCC (2014). Contribution of Working Groups I, II and III to the Fifth Assessment Report of the Intergovernmental Panel on Climate Change.

[57] Guthrie P.D. (1986). Biological methanogenesis and the CO_2_ greenhouse effect. J. Geophys. Res..

[58] Cheng L., Zhu J., Chen G., Zheng X., Oh N.H., Rufty T.W., Richter D.D., Hu S. (2010). Atmospheric CO_2_ enrichment facilitates cation release from soil. Ecol. Lett..

[59] Tokida T., Adachi M., Cheng W., Nakajima Y., Fumoto T., Matsushima M., Nakamura H., Okada M., Sameshima R., Hasegawa T. (2011). Methane and soil CO_2_ production from current-season photosynthates in a rice paddy exposed to elevated CO_2_ concentration and soil temperature. Global Change Biol..

[60] Das S., Bhattacharyya P., Adhya T.K. (2011). Interaction effects of elevated CO_2_ and temperature on microbial biomass and enzyme activities in tropical rice soils. Environ. Monit. Assess..

[61] Inubushi K., Cheng W., Aonuma S., Hoque M.M., Kobayashi K., Miura S., Kim H.Y., Okada M. (2003). Effects of free-air CO_2_ enrichment (FACE) on CH_4_ emission from a rice paddy field. Glob. Chang. Biol..

[62] Qian H., Huang S., Chen J., Wang L., Hungate B.A., van Kessel C., Zhang J., Deng A., Jiang Y., van Groenigen K.J., Zhang W. (2020). Lower-than-expected CH_4_ emissions from rice paddies with rising CO_2_ concentrations. Glob. Chang. Biol..

[63] Alpana S., Vishwakarma P., Adhya T.K., Inubushi K., Dubey S.K. (2017). Molecular ecological perspective of methanogenic archaeal community in rice agroecosystem. Sci. Total Environ..

[64] Wang C., Jin Y., Ji C., Zhang N., Song M., Kong D., Liu S., Zhang X., Liu X., Zou J. (2018). An additive effect of elevated atmospheric CO_2_ and rising temperature on methane emissions related to methanogenic community in rice paddies. Agric. Ecosyst. Environ..

[65] Lindau C.W. (1994). Methane emissions from Louisiana rice fields amended with nitrogen fertilizers. Soil Biol. Biochem..

[66] Zhang L., Song C. (2016). EFFECTS OF NITROGEN ON METHANE PRODUCTION AND OXIDATION AND DISSOLVED ORGANIC CARBON IN A FRESHWATER MARSH. Environ. Eng. Manag. J..

[67] Chen S., Wang Y., Hu Z., Gao H. (2015). CO_2_ emissions from a forest soil as influenced by amendments of different crop straws: Implications for priming effects. Catena.

[68] Kong D., Li S., Jin Y., Wu S., Chen J., Hu T., Wang H., Liu S., Zou J. (2019). Linking methane emissions to methanogenic and methanotrophic communities under different fertilization strategies in rice paddies. Geoderma.

[69] Lou Y., Inubushi K., Mizuno T., Hasegawa T., Lin Y., Sakai H., Cheng W., Kobayashi K. (2008). CH_4_ emission with differences in atmospheric CO_2_ enrichment and rice cultivars in a Japanese paddy soil. Glob. Chang. Biol..

[70] Qian H., Jin Y., Chen J., Huang S., Liu Y., Zhang J., Deng A., Zou J., Pan G., Ding Y. (2022). Acclimation of CH_4_ emissions from paddy soil to atmospheric CO_2_ enrichment in a growth chamber experiment. Crop J..

[71] Guo S., Zhou Y., Shen Q., Zhang F. (2007). Effect of ammonium and nitrate nutrition on some physiological processes in higher plants - growth, photosynthesis, photorespiration, and water relations. Plant Biol..

[72] Walkiewicz A., Bulak P., Brzezińska M., Włodarczyk T.M., Polakowski C. (2012). Kinetics of methane oxidation in selected mineral soils. Int. Agrophys..

[73] Wnuk E., Walkiewicz A., Bieganowski A. (2017). Methane oxidation in lead-contaminated mineral soils under different moisture levels. Environ. Sci. Pollut. Res. Int..

[74] Neue H.-U., Roger P., Zepp R.G. (1994). Climate Biosphere Interactions: biogenic emissions and environmental effects of climate change.

[75] Thanuja G., Karthikeyan S. (2022). Application of soil amendments to enhance soil carbon and biological properties in a paddy field under elevated CO_2_ conditions. Arch. Agron. Soil Sci..

[76] Ye R., Espe M.B., Linquist B., Parikh S.J., Doane T.A., Horwath W.R. (2016). A soil carbon proxy to predict CH_4_ and N_2_O emissions from rewetted agricultural peatlands. Agric. Ecosyst. Environ..

[77] Li S., Xie S., Zhang S., Miao S., Tang S., Chen H., Zhan Q. (2022). Global patterns and controls of the soil microbial biomass response to elevated CO_2_. Geoderma.

[78] Qiu Z., He X., Yu H., Zhu C., Shen W. (2023). Differential responses of soil bacterial communities to elevated CO_2_ between strongly CO_2_-responsive and weakly CO_2_-responsive rice cultivars. Sci. Total Environ..

[79] Cui J., Zheng M., Bian Z., Pan N., Tian H., Zhang X., Qiu Z., Xu J., Gu B. (2024). Elevated CO_2_ levels promote both carbon and nitrogen cycling in global forests. Nat. Clim. Change.

[80] Page M.J., McKenzie J.E., Bossuyt P.M., Boutron I., Hoffmann T.C., Mulrow C.D., Shamseer L., Tetzlaff J.M., Akl E.A., Brennan S.E. (2021). The PRISMA 2020 statement: an updated guideline for reporting systematic reviews. Bmj.

[81] Moher D., Liberati A., Tetzlaff J., Altman D.G., PRISMA Group (2009). Preferred reporting items for systematic reviews and meta-analyses: the PRISMA statement. PLoS Med..

[82] Burda B.U., O'Connor E.A., Webber E.M., Redmond N., Perdue L.A. (2017). Estimating data from figures with a Web-based program: Considerations for a systematic review. Res. Synth. Methods.

[83] Mikhailova E.A., Post C.J., Schlautman M.A., Galbraith J.M., Zurqani H.A. (2018). Usability of soil survey soil texture data for soil health indicator scoring. Commun. Soil Sci. Plant Anal..

[84] Hedges L.V., Gurevitch J., Curtis P.S. (1999). THE META-ANALYSIS OF RESPONSE RATIOS IN EXPERIMENTAL ECOLOGY. Ecology.

[85] Hedges L.V., Tipton E., Johnson M.C. (2010). Robust variance estimation in meta-regression with dependent effect size estimates. Res. Synth. Methods.

[86] Lajeunesse M.J. (2016). Facilitating systematic reviews, data extraction, and meta-analysis with the metagear package for R. Methods Ecol. Evol..

[87] Speidel B. (1992). Effective Care of the Newborn Infant. Arch. Dis. Child..

[88] Midolo G., Alkemade R., Schipper A.M., Benítez-López A., Perring M.P., De Vries W. (2019). Impacts of nitrogen addition on plant species richness and abundance: A global meta-analysis. Global Ecol. Biogeogr..

[89] Cheung M.W.L. (2019). A Guide to Conducting a Meta-Analysis with Non-Independent Effect Sizes. Neuropsychol. Rev..

[90] Cauvy-Fraunié S., Dangles O. (2019). A global synthesis of biodiversity responses to glacier retreat. Nat. Ecol. Evol..

[91] Nakagawa S., Santos E.S.A. (2012). Methodological issues and advances in biological meta-analysis. Evol. Ecol..

[92] Su H., Feng Y., Chen J., Chen J., Ma S., Fang J., Xie P. (2021). Determinants of trophic cascade strength in freshwater ecosystems: a global analysis. Ecology.

[93] Röver C., Rindskopf D., Friede T. (2024). How trace plots help interpret meta-analysis results. Res. Synth. Methods.

[94] Viechtbauer W. (2010). Conducting Meta-Analyses in R with The metafor Package. J. Stat. Software.

[95] Hedges L., Olkin I. (1985). Statistical Methods in Meta-Analysis. J. Educ. Stat..

[96] Egger M., Davey Smith G., Schneider M., Minder C. (1997). Bias in meta-analysis detected by a simple, graphical test. Bmj.

[97] Rosenberg M.S. (2005). The file-drawer Problem Revisited: A General Weighted Method for Calculating fail-safe Numbers in meta-analysis. Evolution.

[98] Mallen-Cooper M., Nakagawa S., Eldridge D.J. (2019). Global meta-analysis of soil-disturbing vertebrates reveals strong effects on ecosystem patterns and processes. Global Ecol. Biogeogr..

[99] Peng S., Kinlock N.L., Gurevitch J., Peng S. (2019). Correlation of native and exotic species richness: a global meta-analysis finds no invasion paradox across scales. Ecology.

[100] Nakagawa S., Lagisz M., O’Dea R.E., Pottier P., Rutkowska J., Senior A.M., Yang Y., Noble D.W.A. (2023). orchaRd 2.0: An R package for visualising meta-analyses with orchard plots. Methods Ecol. Evol..

[101] Liaw A., Wiener M.C. (2007).

[102] Calcagno V., Mazancourt C.d. (2010). glmulti: An R Package for Easy Automated Model Selection with (Generalized) Linear Models. J. Stat. Software.

[103] Rothstein H.R., Sutton A.J., Borenstein M. (2006).

