# Supplementary material for: The impact of elevated CO2 on methanogen abundance and methane emissions in terrestrial ecosystems: A meta-analysis
Source: iScience. 2024 Nov 29;27(12):111504. doi: 10.1016/j.isci.2024.111504 (PMC11697713; doi:10.1016/j.isci.2024.111504)
Supplement: Document S1. Figures S1 [file mmc1.pdf]

iScience, Volume *27*

Supplemental information

The impact of elevated CO_2 on methanogen abundance and methane emissions in terrestrial ecosystems: A meta-analysis

Yiwen Ding, Mingyu Wang, Xiaojuan Du, Xue He, Tianle Xu, Xinyu Liu, and Fuqiang Song

Figures and figure legends:

Figure S1. Egger's regression test for funnel plot asymmetry, related to STAR Methods.

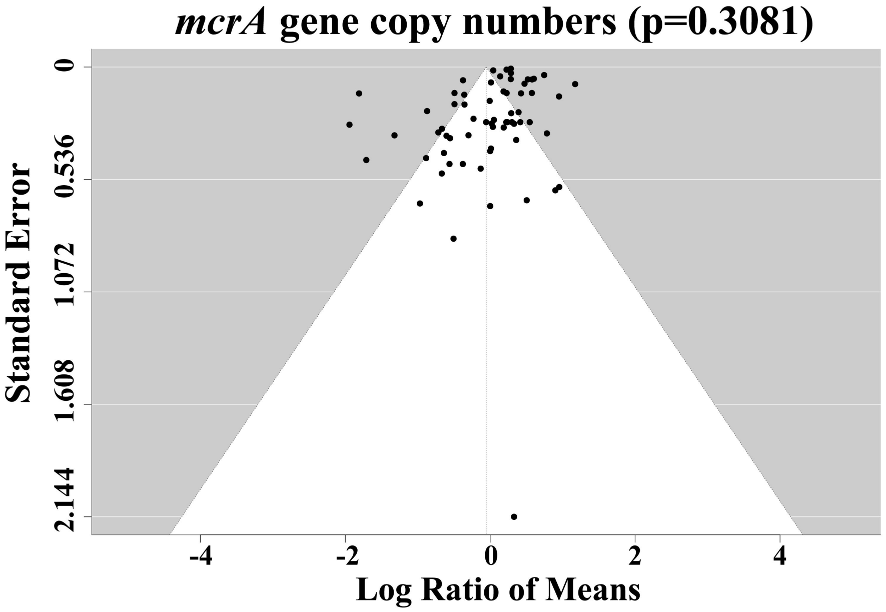

Figure S1. Egger's regression test for funnel plot asymmetry, related to STAR Methods. Effects of eCO_2 on *mcrA* (p=0.3081). The funnel shape was symmetrical at model p-values greater than 0.05 ($P>0.05$). Results were less affected by publication favourability.
